# Re-imagining adherence to treatment from the “other side”: local interpretations of adverse anti-malarial drug reactions in the Peruvian Amazon

**DOI:** 10.1186/s12936-016-1193-x

**Published:** 2016-03-02

**Authors:** Joan Muela Ribera, Susanna Hausmann-Muela, Charlotte Gryseels, Koen Peeters Grietens

**Affiliations:** Departament d’Antropologia, Filosofia i Treball Social Medical Anthropology Research Centre (MARC), Rovira i Virgili University, Avinguda de Catalunya 85, 43002 Tarragona, Spain; Partners for Applied Social Sciences (PASS) International, Tessenderlo, Belgium; Medical Anthropology Unit, Department of Public Health, Institute of Tropical Medicine, Nationalestraat 155, 2000 Antwerp, Belgium; School of International Health Development, Nagasaki University, Nagasaki, Japan

**Keywords:** Treatment adherence, Adverse drug reactions, Community perspectives, Hot and cold theory, Images and metaphors

## Abstract

**Background:**

Patients’ adherence to malaria treatment is a key issue in malaria control and elimination efforts. Previous studies have reported on problems with adherence to anti-malarials, which in part can be related to adverse drug reactions (ADRs) of anti-malarials. However, there has been a relative inattention to the cultural and social aspects of these anti-malarial side-effects and, more broadly, to how cultural representations of body functions may affect people’s behaviour. In this article, an in-depth analysis is presented of the cultural logics underlying local interpretations of adverse drug reactions to anti-malarials in the Peruvian Amazon.

**Methods:**

Ethnographic fieldwork was carried out during two periods of 3 months in 2007 and 2008. Fieldwork was carried out in 10 communities in the department of Loreto, the administrative area corresponding to the Peruvian Amazon. Thirty in-depth interviews of key and general informants, focusing on perceived adverse anti-malarial drug reactions, were carried out in Spanish, recorded, transcribed and analysed.

**Results:**

Informants reported surprisingly elevated problems of adverse drug reactions. Frequent statements about medication that “shocked”, “cut the blood” or provoked “allergic reactions” are difficult to interpret from a biomedical perspective, and only make when considering the underlying cultural logics. The logic of maintaining a ‘temperate’ physical and moral balance by avoiding excesses of ‘hot’ or ‘cold’ or sudden changes of ‘body heat’ can explain the locally constructed adverse drug reactions to anti-malarials.

**Discussion:**

Adherence is a continuous process during which the patient evaluates and re-evaluates the course of his illness and the perceived benefits and risks of the treatment. What counts are the processes, the interpretations and the logics which underlie the decisions to adhere to or to abandon treatment. Adherence can only be adequately addressed if such interpretations are understood and taken into account.

## Background

### Re-imagining malaria through understanding cultural representations

“Far from being an optional “extra”, people need to be our first point of reference when it comes to analysing the barriers to access, product or strategy design; piloting; implementation; feedback, learning and monitoring.” [1]. This quote from the 2015 launched Roll Back Malaria Action and Investment to Defeat Malaria 2016–2030 (AIM)—for a malaria-free world document promotes a vision that actors at all levels engaged in malaria control and elimination potentially agree with: i.e. the need to take the human factor into account—*“the other side”* of malaria interventions. This need is further echoed in the complementary WHO Global Technical Strategy for malaria, mentioning that “context-specific strategies are required to understand better the treatment-seeking behaviours of people in regions with continuing transmission in order to increase demand for treatment, testing and recommended therapy” [2].

Although a focus on community engagement and local context considerations rejoice a revival, there are several aspects that are almost systematically side-lined, including the relevance of cultural representations that guide people’s behaviour. While cultural aspects are often addressed, ‘culture’ is too often reduced to an identification of ‘lack of knowledge’ or ‘wrong knowledge’ that results from ‘local beliefs’ and that explains ‘inadequate practices and behaviours’. The most well-known example in malaria are cases of local cultural interpretations of severe malaria symptoms that guide patients to seek help from traditional medicine, such as ‘*degedege’* in Tanzania [3, 4], or *jinne* (spirits) attacks in the Gambia [5]. Such studies have certainly been very useful for culturally adequate messaging in many information campaigns, with quite a success for changing behaviours. However, it is also clear that descriptions of isolated cultural representations are only the ‘mushrooms’ that are visible on the surface while the deeper cultural structure is much more complex, representing the ‘*mycelium’*, the invisible and connected fabric of growing threads beneath the surface. Exploring this ‘*mycelium* of culture’ implies studying locally prevailing ‘logics’ and disentangling the way local knowledge is structured, on the basis of these logics. The term ‘logic’ has been used mostly by French social scientists [6] to refer to the structure of meaning that links and provides internal coherence to what people say and do, i.e. to shared knowledge and practices. Such analysis, that goes beyond the momentary collecting or counting ‘mushrooms’ (i.e. isolated cultural representations) to scrutinize the contextual and historically forged ‘*mycelium’* studies slow-changing processes and permits us to understand the underlying logics that help to explain the meanings behind observed practices. Understanding the structure of meaning, as they are shared in a community, paves the way for formulating communication messages that make sense to people in the local context, and allows to propose strategies aligned with people’s culture and understanding. It is this deep analysis, this search for understanding the underlying logics that creates new views on local knowledge and related practices. As social scientists, we are invited to re-imagine malaria and to understand it not in biomedical terms, but in its cultural representations. The example of adverse drug reactions (ADRs) in the Peruvian Amazon, which finds it origin from intricate findings from the field led to the exploration of the logics, the *mycelium*, that helps us to explain paradoxical responses and practices from patients.

### The paradox of patients’ response to anti-malarial treatment

Although adverse drug reactions (ADRs) are known to affect adherence [7], surprisingly there has been a relative inattention to the cultural and social aspects of these side-effects. This article presents an in-depth analysis of the reasons why people do not adhere to the prescribed 7 days anti-malarial drug regimen for *Plasmodium vivax* in the Peruvian Amazon. An earlier paper [8], had reported an adherence rate of 62.2 % to the full 7 day primaquine course for *P. vivax* in the Peruvian Amazon, with the majority of patients abandoning treatment after 3 days despite it being free of charge. Due to the marked increase in malaria incidence in the study area over the past decade, treatment adherence to primaquine (PQ) for the radical cure of *P. vivax* infection is more than ever a major public health concern in the Peruvian Amazon. A high percentage of these infections are suspected to be relapses or recrudescences due to either treatment failure or poor treatment adherence. Though rarely fatal, the persistent nature of the *P. vivax* in the human liver (hypnozoites) can produce repeated relapses for years after the initial infection, making adherence particularly relevant to eliminate the latent hypnozoite reservoir after symptoms abate. Contrary to expectations, only 10.8 % of the surveyed vivax malaria patients associated discontinuation of treatment with their improved health status after 3 days. Almost half of the respondents (48.7 %) related non-adherence to the treatment to the adverse side effects of the drugs. Asked about their personal experience with anti-malarial treatment, two out of three respondents (69.9 %) stated that the medication “shocked” (chocó). Nearly the same proportion (61.2 %) mentioned that anti-malarials provoked allergic reactions. In contrast to the medically described ADRs of CQ (gastrointestinal problems, stomach ache, itch, headache, nightmares and blurred vision) and to a lesser extent of PQ (nausea, vomiting and stomach cramps, headache, visual disturbances and intense itching), the reported ADRs in the Peruvian Amazon are difficult to interpret without understanding the underlying cultural logics presented in this paper.

## Methods

### Study site and population

#### Site

The study area consists of the communities along the road from Iquitos to Nauta, in the department of Loreto, the administrative area corresponding to the Peruvian Amazon. All communities are situated in the vicinity of the Itaya (East) and Nanai (North-West) Rivers. These communities, with an estimated population of 5300 people, have relatively recently been established.

#### Population

The majority of the inhabitants are poor farmers, mainly ‘*mestizos’—*referring to all Peruvians that cannot be clearly identified as belonging to any ethnic minority population. Local subsistence strategies include slash and burn agriculture, fishing, hunting, and small scale charcoal production. Occasionally, people also engage in fish farming, logging, small commercial activities and paid employment as grounds’ keepers or cultivators for institutions, farms and enterprises belonging to wealthier Iquitos’ residents. Further setting specific details are described in a previous article [8].

#### Malaria treatment guidelines

In 2001, the Peruvian National Malaria Control Program established the current first-line treatment of 3 days chloroquine (CQ) and 7 days primaquine (PQ). The anti-malarial drugs can be purchased free of charge in the health centres along the road, in Paujil and Cahuide villages. Patients are requested to visit the health centres three times. At the first visit, a diagnostic test is carried out, and treatment (CQ and PQ) is provided for the first 3 days. Only if the patient lives far away, the full treatment for the 7 days is provided. At the second visit, the patient receives primaquine for the remaining 4 days in order to complete treatment. The third is a control visit to clinically evaluate cure.

### Study design

The present research was part of a mixed methods study using a sequential [QUAL → quan → qual] triangulation design [9] to get an in-depth understanding of factors related to the (non-) adherence to the 7 day CQ-PQ regimen of microscopically confirmed *P. vivax* malaria cases. Results presented here are based on the in-depth [QUAL] strand of the study. Results of the overall study are published elsewhere [8].

### Data collection

Ethnographic fieldwork was carried out during two periods of 3 months in 2007 and 2008, triangulating participant observation, informal interviews and in-depth interviews. Fieldwork was carried out in 10 communities: El Dorado, 13 de Febrero, Nuevo Horizonte, Ex-Petroleros, 12 de Abril, Cahuide, Villa Buen Pastor, Paujil, 24 de Junio, and El Triunfo. Thirty in-depth interviews of key and general informants, focusing on perceived adverse anti-malarial drug reactions, were carried out in Spanish, recorded and transcribed. The interviews were held in the selected above-mentioned locations, representing a sufficient number of respondents to get a coherent understanding of the local context and social setting. Informal conversations were not recorded. Notes were kept of the most relevant observations and conversations.

Sampling for all informal and formal interviews was purposive. Informants were selected according to relevant variables such as gender, age, subsistence strategy, locality, adherence, etc. to allow for maximum variation in the sample. Sampling techniques included respondent-driven or snowball sampling to improve data reliability.

### Data analysis

Qualitative data were entered and analysed using N/Vivo Qualitative Analysis software (QSR International Pty Ltd., Cardigan, UK). The analysis was a flexible and iterative process; preliminary data obtained from different techniques were entered and analysed to generate temporary results. Further research was then conducted to confirm or refute these temporary results until saturation was reached and data could be theoretically supported.

### Ethical clearance

The study was approved by the Ethical Committee of the University of Antwerp, Belgium and the Ethical Review Committee of the Universidad Peruana Cayetano Heredia, Lima, Peru. During field work, all interviewers followed the Code of Ethics of the American Anthropological Association (AAA). As proposed by the AAA, all interviewees were informed before the start of the interview about project goals, the topic and type of questions, their right to refuse being interviewed, interrupt the conversation at any time, and withdraw any given information during or after the interview, and the intended use of the results for scientific publications and reports to health authorities.

## Results

### Malaria and the logics of treatment in Iquitos

#### *‘*Hot and cold’ in anti-malarial treatment

A typical malaria attack consists of intermittent cold (shivering), hot (fevers and vomiting), and sweating stages. In Iquitos, malaria is considered a ‘hot’ illness with ‘cold’ phases. ‘Hot’ and ‘cold’ do not refer to actual temperature, but to the *qualities* of things. They are used to denominate illnesses as much as food and beverages, drugs and plants, and apply to persons according to age and gender, to activities and bodily states.

Traditional treatments against malaria combine ‘hot’ and ‘cold’ plants. Among the ‘hot’ and ‘very bitter’ plants is *remo caspi* or paddle tree, commonly used against fevers and malaria throughout the Amazon rainforest. Other medicinal plants such as *abuta*, *hausai*, *mullaca, guisador* (turmeric), *cedro rosa* (Spanish cedar), verbena, *pifuayo, sinamillo* are all considered ‘hot’. The pharmaceutical anti-malarials chloroquine and primaquine are also classified as ‘hot’ and ‘bitter’. Among the ‘cold’ herbal remedies for malaria are mauve, lime leaves, and lime juice. To reduce malaria fevers, people take warm baths containing ‘cold’ herbs or bathe in the river. In addition, people take the commercial *Eno fruit salt*, papaya, grapefruit and other ‘fresh’ fruit or ‘refreshing’ fish soup to counterbalance the effect of ‘hot’ and ‘bitter’ plants and drugs. ‘Cold’ remedies are also used to ‘cool down’ the fever and to alleviate stomach burning due to malaria or pharmaceuticals.

People who have suffered from malaria express the sensation of the cold stage with phrases like *“it made me shake”, “I felt cool, I went home and even more cold overcame me and I wrapped myself in a blanket”,* or *“so much shivering you get cramps”. Remo caspi*, *abuta* and other bitter plants are taken before the shivering starts, *“when you feel that your body is worsening and you will get malaria, then you take it”, “when you feel the cold, this is when you take the bitter plant”*. Because a bitter plant is considered a ‘hot’ therapy, the body must be cooled down (for example by taking a bath) at this time and intake of ‘hot’ foods as well as exposure to intense cold should be avoided. Some informants agreed to combine anti-malarials with ‘bitter’ herbs, but most said that a mixture was dangerous due to an excess of ‘hot’ remedies. They stated that a malaria patient should take either one or the other, or take them consecutively, first drugs and then herbs, or vice versa.

#### The perception of relapses: excess of heat

The local term for recurrence is *‘rebrote’*. In biomedical terms, *‘rebrote’* corresponds to relapse or rebound malaria. A relapse refers to a reactivation of latent parasites in the liver cells that can occur after years; rebound malaria occurs due to an insufficiently cleared malaria infection. The informants usually speak of *‘rebrote’* when they refer to a recurrence of illness within a few days, weeks or months after a malaria episode. The local interpretation of *‘rebrote’* follows the logic of the ‘hot and cold’ theory rather than the biomedical explanation. Hot food and beverages, such as pork meat or alcohol, are seen as responsible for the activation of a *‘rebrote’* or relapse, as the following quote clearly shows:

*…Then malaria leaves you, you eat pork meat and it is a rule that it recurs* (…) *the same with masato* (a fermented yucca drink) *and if it rains on you, it is even worse* (male farmer, age 43).

*The perception* that relapsed or ‘*rebrotada*’ malaria is more virulent than the first episode is common. According to some informants relapsed malaria can even cause death. In the questionnaire, more than half (27) and almost one quarter (10) of the 45 respondents who mentioned a reason for *‘rebrote’* answered ‘eating greasy food’ and ‘drinking alcoholic beverages’, respectively. Only one respondent mentioned ‘incomplete treatment’ as the reason. In addition, several in-depth interviews confirmed the common perception that ‘incomplete treatment’ may keep the illness dormant, while recurrences are provoked by an excess of hot food or beverages. One informant said:

*Some came with malaria, they ate something greasy, and sometimes they are not aware, maybe they eat a ‘juane’* (a typical dish of the Peruvian jungle that contains rice, chicken, and a variety of ‘hot’ herbs and leaves), *thus again they get fever, it comes with vomiting, they get diarrhoea, again but more fiercely* (male village health worker and farmer, age 28).

The perception of ADRs: ‘shocks’.

The same logic of excess of heat and sudden changes are perceived problematic for malaria patients. A health worker explained:

*If you have malaria, you cannot take drugs and strong things, alcohol, pork meat, chicken meat, masato, because this shocks.* (female village health worker and shopkeeper, age 33).

The feeling of ‘shock’ is described as a sudden impact, a stroke, or an ‘attack’ that affects the head, the stomach, and sometimes the liver. Informants report a sharp pain, dizziness and nausea as a result of *‘choque’*.

Although anti-malarials are accepted as the correct treatment, they are also feared because they ‘shock’. This is one reason for abandoning treatment. One informant explained:

*The pills shock me* (‘me chocan’)*, it makes me feel dizzy when I take these pills, this is why I did not want to take them* (male farmer, age 62).

Another informant explained that she reduced the dosage of anti-malarials.

*With primaquine, the fever stopped, but my body had become very weak, very tired, I hardly walked five meters and I got tired, therefore when I sat down and waited uuum!… easily you fall down very weakly… thus in the end I reduced the drug to one daily dose* (female farmer, age 22).

According to local explanations, the drugs by themselves can ‘shock’. They can ‘shock’ even more when taken with ‘hot’ foods and beverages such as alcohol, *masato* and pork meat, or together with ‘bitter’ herbal remedies.

To prevent recurrences, informants reported to attempt to avoid ‘shocks’, or to counterbalance its effects, by ‘cooling down’ the body with ‘cold’ mauve infusions, lemonades, or baths. Beyond food, beverages, plants, and drugs, ‘shock’ also relates to the constitution of the body. Weak persons, who have ‘low blood’ (which might refer to anaemia), either by nature or due to illness or a combination of both, are said to be more susceptible to ‘shock’. Body constitution is also relevant for recurrences, where an accumulation of ‘hot’ elements and behaviours is central. A health worker reported:

*“And you know that when the body is weak, when you work and you don’t eat well, and on top of that you are exposed to the sun, and it* (malaria) *comes again, just more complicated, then you keep repeating, I mean you get well during 7*  *days, and between seven and 15* *days you get it again, because you were not resting* (…) *with the masato again it starts to go up* (relapsing malaria) (…) *sometimes you take liquor, they* (the friends) *come to have some drinks, oh dear, and you already feel like a stroke for not having adhered to treatment”* (male village health worker and farmer, age 28).

As such, alcohol plays an important role in treatment abandonment. It was often said that many people *“prefer drinks to treatment”*, referring to hard drinkers who abandoned treatment *“in order to avoid the shock”* as they could not stop consuming alcohol. One informant recalled a man who *“did not finish his treatment, took strong masato, and in the afternoon he was dead”* (male farmer, age 59).

In sum, in Iquitos, ADRs to antimalarial treatments are expressed by using the concept of ‘shock’. But ‘shock’ is more than the felt impact when a drug is taken. Informants’ narratives portray how ‘shock’ refers to a bodily experience, explained by the logics of ‘excesses’ and ‘contrasts’ that, in turn, form part of the hot and cold theory. Fears to ‘shocks’, as represented locally in the sense of these common logics, can lead to an interruption of treatment, particularly during the ‘hot phase’ that follow the ‘cold phase’, when ‘hot’ treatments are perceived to be potentially dangerous, due to the effects of ‘excesses’ (i.e. hot phase plus hot treatment) [8].

## Discussion

### The model behind perceived ADRs: the ‘hot and cold’ theory in Latin America

In Latin America the ‘hot and cold’ theory permeates popular models of health and illness. Derived from humoral theory, it was brought to America by the Spanish and Portuguese during colonial times and remained the dominant paradigm in medicine until its decline in the nineteenth century [10, 11]. While it is uncertain whether these imported concepts of humoralism converged with similar autochthonous concepts or whether they were newly introduced to local health and illness models, it is clear that these principles were well integrated into the local world-views [12]. However, several authors have pointed out that in Latin America, the ‘hot and cold’ theory is more dynamic and pragmatic than it was in Europe [13, 14].

It is misleading to describe the ‘hot’ and ‘cold’ principles as a dichotomy because the theory refers to a *continuum* with different *grades* rather than clear-cut categories [10]. Even in historical accounts, in the early eighteenth century, Brother Pedro de Montenegro [15] remarked that the pairs of ‘hot/cold’ and ‘dry/wet’ qualities are each divided into four grades, from least to most, with the first being the most benign, and the last the most dangerous grade. Current popular models include intermediary categories of ‘fresh’ and ‘warm’ which, applied to drugs, plants, food, and beverages, are very important for treatment. By understanding the ‘hot/cold’ system as a *continuum,* an illness can *transmute* from one state to another [16]. Adding up hot elements, even gradually and with small increments of heat, may finally lead to pathological *excess*, like the last straw that breaks the camel’s back.

Arroyo Laguna and colleagues [17] reported similar results from Peru where respondents mentioned that intake of hot foods, combined with environmental heat and heavy workload produced illness. Following the same logic, Logan’s study in Guatemala [18] described the perceived detrimental effects of an excess of ‘cold’ when a ‘cold’ illness is treated with a ‘cold’ drug like penicillin. To regain health one has to restore the bodily balance: a ‘cold’ illness needs to be balanced with warm beverages, baths, medicines, and foods. Vice versa, a ‘hot’ illness requires to be cooled down with cold beverages, baths, medicines, and foods.

A sudden change from ‘hot’ to ‘cold’ or vice versa can provoke a ‘shock’. For instance, passing from a hot to a cold place, exposure to rain while sweating at work, a cold bath after exposure to the sun are activities that are perceived to cause illness, as a result of the ‘shock’ produced in the body. To avoid this, the body needs to be *gradually* accommodated, for example, by first wetting one’s body before entering a hot or cold bath. ‘Cold’ and ‘hot’ must harmonize in order to produce a ‘temperate’ physical and moral balance and to achieve organic and emotional well-being [14].

### Why re-imagining treatment adherence from people’s perspective matters—the hot and cold theory in the context of malaria elimination

Although intensive community-based interventions have shown substantial improvements [19, 20], adherence remains problematic even when drug supply is good [21]. Besides structural, behavioural and other relevant factors that may hinder adherence, social representations of medicines play a key role. Studying perceived efficacy and side effects of anti-malarials in Tanzania, Kamat [22] found that the cultural and social meanings attributed to drugs, i.e. cultural constructions of certain brand names and the perceived efficacy of different anti-malarials administered at public health facilities have a major impact on the health seeking itineraries of malaria patients. Public, free of charge treatments at local health facilities are not always preferred options precisely because of perceived drug efficacy and the cultural constructions of certain drugs [21, 22].

In Iquitos, an unexpectedly high percentage of respondents reported to have experienced ADRs, namely ‘shocks’ (70 %) and ‘allergies’ (61 %). These ADRs were seen as the principal reason for abandonment of malaria treatment. The importance of perceived adverse effects was also a main reason for non-adherence in a study carried out in the province of Esmeraldas in Ecuador, in which adherence was estimated at 65.9 % based on interviews with clinically confirmed vivax and falciparum malaria patients [23]. A qualitative study in Piura and Tumbes in Peru also cited the quick lessening of symptoms and the perceived adverse effects of treatment as the main elements for diminishing adherence [24].

As health in the Peruvian Amazon is understood to be a balance between ‘hot and cold’, intake of ‘hot’ anti-malarials needs to be compensated by other components (diet, herbal remedies, baths, etc.) that restore health. The local construction of ADRs is part of this holistic perspective, which does not take drug reactions per se into consideration, but integrates them into a system of excesses and contrasts of ‘hot’ and ‘cold’. This local model includes five principles that have practical implications for adherence: (i) If treatment is abandoned, the illness remains ‘dormant’. Eating pork or drinking alcohol increases the risk that malaria ‘comes up’ or ‘relapses’; (ii) the ‘hot’ quality of anti-malarials poses a risk of ‘shock’, ‘allergies’, and other adverse reactions; (iii) this risk increases substantially if the patient ingests food, drinks, medicinal plants, or other remedies considered ‘hot’ (following the logic of ‘excess’) or ‘cold’ (following the logic of ‘contrast’), if the patient performs activities which ‘overheat’ the body, or if he gets exposed to excessive heat or sudden cold; (iv) the risk is reduced by tempering the body with ‘refreshing’ teas, foods, drugs, or baths; and (v) the person’s constitution increases or reduces the risk to suffer from adverse reactions.

The implications of the local model for adherence can be two-sided. While it is true that the ‘hot and cold’ theory can prompt patients to interrupt treatments, the logic of balance that underlies the theory permits re-imagining treatment beyond dominant images in malaria interventions. Current approaches are grounded in the war metaphor (the war or the fight against malaria) and focused on biomedical-technological solutions [25], which make anti-malarial campaigns pathogen and vector-centred. The implications of the war metaphor as used in the malaria field, are that the target is an objectified disease, anti-malarials are ‘weapons’, and experts (researchers, medical personnel) are the ‘fighters’, leaving aside people’s experiences and social realities. In contrast, the ‘body in balance’ metaphor, linked to the ‘hot and cold’ theory, as used in Iquitos and many other parts of the world, is clearly person-centred. Like the disease, medicines affect each person differently, according to their age and sex, to what they eat and drink, to their work and other activities. Anti-malarial drugs can heal, but they can can produce or increase imbalance and, therefore, disease. In this sense, the local model of malaria and its treatment in Iquitos is closer to patient/people-centred approaches than anti-malarial campaigns. A patient-centred approach includes not only the use of efficacious and quality medicinal products to target the disease, but takes a person’s circumstances, life conditions and feelings into account [26].

The shift from pathogen to patient-centred approaches has implications for health promotion because a focus on people necessarily requires a horizontal, participatory approach that goes beyond designing health messages to encourage people to accept and adhere to treatments in order to ‘fight a disease’. Formative research is a useful way to elaborate health promotion strategies together with the communities, and as such respond to real needs [27–29]. With regard to adherence promotion in the Peruvian context, a formative research approach would consider, for example, the interconnected nutritional, recreational, climatic, work conditions and so on, and identify, together with the affected communities, the best ways to commensurate effective treatments with local perceptions and experiences.

Moreover, the ‘hot and cold’ theory can serve as a valuable entry point for a systemic response to the disease accounting for its social and ecological determinants. Being multicausal and systemic, dynamic and relational, this perspective opens a window of opportunity for a multisectorial and transdisciplinary approach uniting public health goals and community needs.

## Conclusion

Increased efforts of malaria control and elimination have focused on improved access to diagnostics and drugs, but adherence to treatments remains under-researched. Adherence is not a yes/no decision but a continuous process during which the patient evaluates and re-evaluates the course of his illness and the perceived benefits and risks of the treatment. The processes, the interpretations and the cultural logics which underlie the decisions to adhere to or to abandon treatment are crucial to take into account in malaria elimination efforts.

Re-imagining malaria, the overarching topic of the 2014 workshop at LSHTM and the launch of the thematic series [30] of which this article forms part, reflects on different perspectives ‘to see’ malaria, and goes deep into the analysis of how they are locally constructed. Using the example of drug adherence, this article examined the perspectives of the ‘other side’, the patients’ response, and unveils people’s fears for taking treatments and reasons for discontinuation.
